# Biallelic mutations in *CDC20* cause female infertility characterized by abnormalities in oocyte maturation and early embryonic development

**DOI:** 10.1007/s13238-020-00756-0

**Published:** 2020-07-14

**Authors:** Lin Zhao, Songguo Xue, Zhongyuan Yao, Juanzi Shi, Biaobang Chen, Ling Wu, Lihua Sun, Yao Xu, Zheng Yan, Bin Li, Xiaoyan Mao, Jing Fu, Zhihua Zhang, Jian Mu, Wenjing Wang, Jing Du, Shuai Liu, Jie Dong, Weijie Wang, Qiaoli Li, Lin He, Li Jin, Xiaozhen Liang, Yanping Kuang, Xiaoxi Sun, Lei Wang, Qing Sang

**Affiliations:** 1 Institute of Pediatrics, Children’s Hospital of Fudan University and the Shanghai Key Laboratory of Medical Epigenetics, the International Co-laboratory of Medical Epigenetics and metabolism, Ministry of Science and technology and Institutes of Biomedical Sciences, State Key Laboratory of Genetic Engineering, Fudan University, Shanghai, 200032 China; 2 Center of Assisted Reproduction, Shanghai East hospital, Tongji University, Shanghai, 200120 China; 3 The Reproductive Medical Center of Xiangya Hospital, Central South University, Changsha, 41008 China; 4 Reproductive Medicine Center, Shaanxi Maternal and Child Care Service Center, Xi’an, 710069 China; 5 NHC Key Lab of Reproduction Regulation (Shanghai Institute of Planned Parenthood Research), Fudan University, Shanghai, 200032 China; 6 Reproductive Medicine Center, Shanghai Ninth Hospital, Shanghai Jiao Tong University, Shanghai, 200011 China; 7 Shanghai Ji Ai Genetics and IVF Institute, Obstetrics and Gynecology Hospital, Fudan University, Shanghai, 200011 China; 8 Key Laboratory of Molecular Virology & Immunology, Institute Pasteur of Shanghai, Chinese Academy of Sciences, Shanghai, 200031 China; 9 Bio-X Center, Key Laboratory for the Genetics of Developmental and Neuropsychiatric Disorders, Ministry of Education, Shanghai Jiao Tong University, Shanghai, 200030 China; 10 State Key Laboratory of Genetic Engineering and Collaborative Innovation Center for Genetics and Development, School of Life Sciences, Fudan University, Shanghai, 200438 China; 11 Zhuhai Fudan Innovation Institute, Zhuhai, 519000 China; 12 Shanghai Center for Women and Children’s Health, Shanghai, 200062 China

**Dear Editor,**

Previously, the Mendelian phenotypes in human oocyte maturation arrest, fertilization failure and early embryonic arrest, are largely underestimated. In recent years, “missing” Mendelian phenotypes and genes in these processes are beginning to be uncovered by us and others (Huang et al., 2014; Alazami et al., 2015; Feng et al., 2016; Xu et al., 2016; Chen et al., 2017; Sang et al., 2019). However, the genetic basis for majority of patients resulting from abnormalities in these phenotypes remains to be elucidated.

The cell division cycle 20 (CDC20, HGNC:1723) is the co-activator of anaphase-promoting complex/cyclosome (APC/C) during mitosis, and plays a role in maintaining the genome by regulating spindle assembly checkpoints (Musacchio and Hardwick, 2002). In oocytes, the activation of APC/C by CDC20 is a key step in homologue disjunction and in transition from meiosis I to meiosis II (Jones, 2011). CDC20 is therefore an essential component of the mammalian cell cycle mechanism regulating both miotic and meiotic exit. Although *CDC20* is an extensively studied gene, until now, no solid evidence has been provided to establish the causal relationship between *CDC20* mutations and human diseases. The only report was an association study relating *CDC20* mutations with idiopathic azoospermia (Li et al., 2017).

Here, we identified biallelic *CDC20* mutations in five infertile individuals with oocyte maturation arrest, fertilization failure, and early embryonic arrest. We investigated the effects of the corresponding mutations in cell lines and mouse oocytes and explored a potential therapeutic treatment by direct *CDC20* cRNA injection.

The probands in families 1 and 2 had normal menstrual cycles and had been diagnosed as primary infertility with unknown reasons for several years (Table S1). Both of the probands came from consanguineous families (Fig. 1A). The probands in family 1 had undergone two failed *in vitro* fertilization (IVF) attempts and one failed intracytoplasmic sperm injection (ICSI). In her ICSI cycle, eleven oocytes were obtained, and ten oocytes were arrested at the germinal vesicle (GV) or metaphase I (MI) stage (Fig. 1B), only one was matured and fertilized, but was arrested at three-cell stage (Table S1). The proband in family 2 had undergone two failed ICSI cycles. All of the retrieved oocytes were arrested at the MI stage (Table S1). In summary, both two probands had phenotypes of oocyte maturation arrest in their ICSI attempts. Whole-exome sequencing and homozygosity mapping was performed (Fig. S1). By a recessive inheritance model, we identified two homozygous missense mutations (c.683A>G, p.Tyr228Cys and c.1316T>G, p.Leu439Arg) in *CDC20*, respectively (Fig. 1A). Because the parents of both probands were unavailable, in order to rule out the possibility that the homozygous status of *CDC20* resulted from the deletion of one allele, we performed a copy number variation (CNV) analysis. There was no CNV for *CDC20* in the probands of both families, which confirmed that the mutations in the two individuals were indeed homozygous (Fig. S2A and S2B).

**Figure 1:**
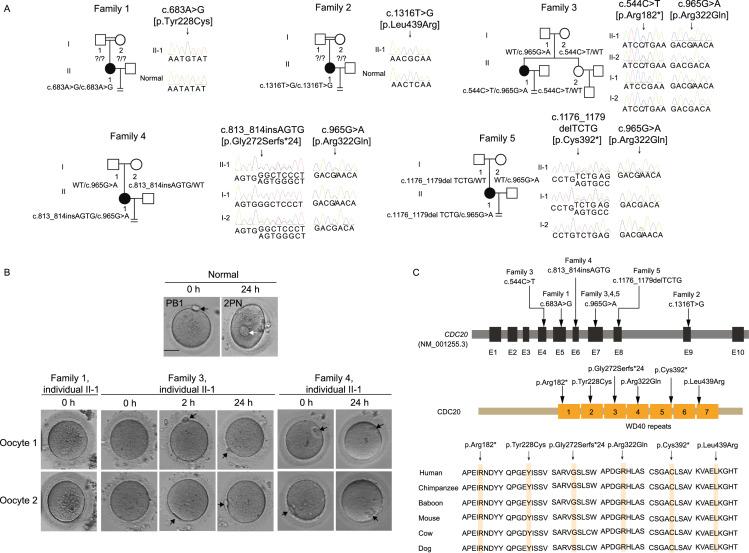

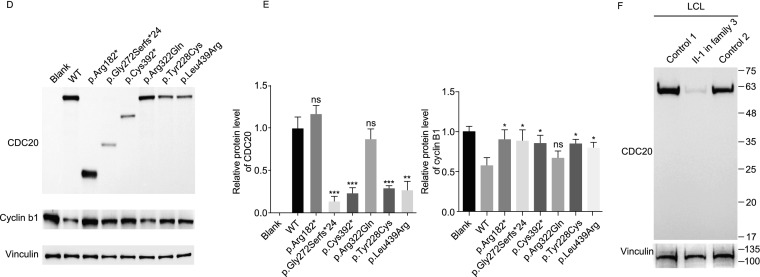
**Identification of mutations in** ***CDC20*** **and effects of the mutations on protein level of CDC20 and cyclin B1.** (A) Five pedigrees presented with abnormalities in oocyte maturation, fertilization and early embryonic development. Question marks indicate unavailable DNA samples, double lines denote consanguineous marriage, the equal sign denote infertility. (B) The morphology of normal and affected individual oocytes at 0 h, 2 h and 24 h after retrieved. The black arrows indicate the first polar body (PB1), and the white arrow in the normal oocyte indicates the pronuclei. Scale bar = 40 μm. (C) Locations and conservation of mutations in *CDC20*. (D) The effects of the mutations on CDC20 and cyclin B1 protein level by Western blot in transfected CHO cells. (E) Left: Quantitation of blank, wild type (WT) and mutant CDC20 protein level in Fig. 1D. The data are shown as means and SD. **P < 0.01, ***P < 0.001, ns, not significant. Right: Quantitation of cyclin B1 protein level of overexpression the WT and mutant CDC20 in Fig. 1D. The data are shown as means and SD. *P < 0.05; ns, not significant. The experiment was performed with three independent biological replicates yielding similar results. (F) The effects of the mutations of p.Arg182* and p.Arg322Gln on CDC20 protein level by Western blot in LCLs of individual II-1 in family 3

Mutational screening of *CDC20* were pursed in a large cohort of 1,250 infertile individuals with abnormalities in oocyte maturation, fertilization and early embryonic development by whole exome sequencing. Additional biallelic mutations in *CDC20* were detected in another three individuals from families 3–5 (Fig. 1A). The compound heterozygous mutations in *CDC20* in the affected individual in family 3 consisted of a missense mutation c.965G>A (p. Arg322Gln) and a nonsense mutation c.544C>T (p. Arg182*). The proband in family 4 carried a compound heterozygous mutation consisting of a missense mutation c.965G>A (p.Arg322Gln) and a 4 bp insertion c.813_814ins AGTG (p.Gly272Serfs*24). The proband in family 5 carried a compound heterozygous mutation consisting of a missense mutation c.965G>A (p.Arg322Gln) and a 4 bp deletion c.1176_1179del TCTG (p.Cys392*). Sanger sequencing were performed to confirm the mutations in these three families (Fig. 1A). Information about the mutations is shown in Fig. 1A and Table S2, and the positions of the mutations and their conservation in different species are shown in Fig. 1C. In family 3, oocytes were immature when retrieved. Most of immature oocytes could develop into the first polar body (PB1) oocytes after 2 hours’ *in vitro* culture, but showed fertilization failure or early embryonic arrest. The proband in family 4 and family 5 had phenotypes of fertilization failure or early embryonic arrest. The specific clinical information is indicated in Fig. 1B and Table S1.

In transfected Chinese hamster ovary (CHO) cells, the missense mutations p.Tyr228Cys and p.Leu439Arg resulted in a reduction in CDC20 protein level, while mutations p.Arg182*, p.Gly272Serfs*24, and p.Cys392* resulted in truncated proteins (Fig. 1D and 1E). For mutation p.Arg322Gln, though there was no obvious effect on CDC20 protein level in CHO cells, the Western blot analysis of lymphoblastoid cell line (LCL) of the affected individual II in family 3 with a missense mutation c.965G>A (p. Arg322Gln) and a nonsense mutation c.544C>T (p. Arg182*) showed significantly reduced protein level (Figs. 1F and S3A). In addition, the mRNA expression of *CDC20* in the LCLs of individual II-1 in family 3 and in the granulosa cells (GCs) of individual II-1 in family 4 were also reduced significantly (Fig. S3B and S3C). We also explored the effects of the mutations (c.683A>G, c. 813_814ins AGTG, c.1176_1179del TCTG, and c.1316T>G) on mRNA expression in transfected CHO cells, and the results showed that all these four mutations caused significantly reduced mRNA expression (Fig. S4). All these results indicate that mutations in *CDC20* lead to unstable protein and degraded RNA *per se*. During the metaphase to anaphase transition, APC/C is activated through the release from CDC20 inhibition, and this leads to the breakdown of cyclin B1 (HGNC:1579) (Nasmyth and Haering, 2005). We therefore determined the effects of *CDC20* mutations on cyclin B1 degradation. As shown in Fig. 1D and 1E, overexpression of wild-type CDC20 significantly decreased the endogenous protein level of cyclin B1, while most of mutations affected the degradation of cyclin B1.

We next investigated the effects of the mutations on human CDC20 localization in mouse oocytes. For the three missense mutations (p.Tyr228Cys, p.Arg322Gln, p.Leu439Arg), CDC20 showed normal kinetochore localization as the wild-type. In contrast, CDC20 failed to localize to the kinetochore for the other three nonsense or frameshift mutations (p.Arg182*, p.Gly272Serfs*24, p.Cys392*) (Fig. 2A), indicating these are loss-of-function mutations.

**Figure 2:**
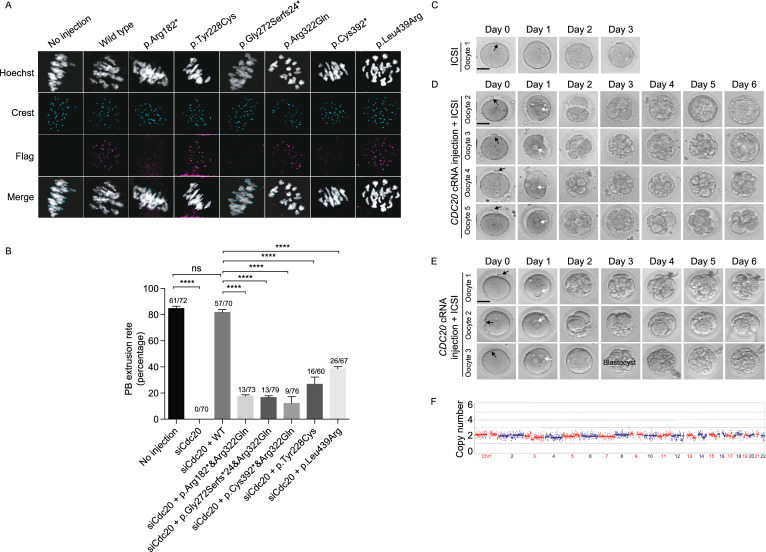
**Effects of mutations on CDC20 localization and function in mouse oocytes and phenotypic rescue by** ***CDC20*** **cRNA injection into oocytes from the proband of family 3 and family 4.** (A) Localization of WT and mutant FLAG-tagged CDC20 in mouse oocytes. Hoechst and Crest were used to label the DNA and kinetochores, respectively. (B) The effects of the mutations on the rescue of PB1 extrusion in *Cdc20* knockdown mouse oocytes with phenotype of MI arrest. The number of oocytes used are listed on top of the column. Three independent experiments were performed. ****P < 0.0001; ns, not significant. (C) One PB1 oocyte of individual II-1 in family 3 underwent ICSI without *CDC20* cRNA injection was used as control. Day 0 indicates the time point at 4 h after ICSI. The black arrowhead indicates the polar body. Scale bar = 40 μm. (D) Four retrieved PB1 oocytes of individual II-1 in family 3 were injected with *CDC20* cRNA and cultured for 4 h and then used for ICSI. Oocytes were monitored for 6 days after ICSI. The white arrows indicate the pronuclei. (E)Three retrieved PB1 oocytes of individual II-1 in family 4 were injected with *CDC20* cRNA and cultured for 4 h and then used for ICSI. (F) Trophectoderm cells from the blastocyst embryo of individual II-I in family 3 were collected and sequencing for chromosomal copy number variation analysis

Knockdown of the *Cdc20* gene exclusively blocks PB1 extrusion in mouse oocytes and the PB1 extrusion can be rescued by injecting *Cdc20* cRNA into the oocytes (Reis et al., 2007). To further explore the effect of mutations on CDC20 function in oocytes, we first knocked down the endogenous *Cdc20* in mouse oocytes by using *Cdc20* siRNA, and we observed an MI arrest phenotype that could be rescued by supplementation with human wild-type *CDC20* cRNA (Fig. 2B). We then performed a rescue experiment using patient-derived mutant cRNAs in the same way. Compared with wild-type, all mutations significantly reduced the ability of CDC20 to rescue PB1 extrusion (Fig. 2B). These results indicate the overall impaired effect on CDC20 function of homozygous and compound heterozygous mutations.

We then explored a potential therapeutic treatment for two patients by *CDC20* cRNA injection. For the proband in family 3, compared with control, all four oocytes injected with *CDC20* cRNA were successfully fertilized as indicated by the formation of two pronuclei on day 1, and two of the oocytes developed into blastocysts on day 6 (Fig. 2C and 2D). For the proband in family 4, all three oocytes injected with *CDC20* cRNA were successfully fertilized on day 1, and one developed into an eight-cell stage embryo (Fig. 2E). Preimplantation genetic screening showed that one of blastocysts in family 3 had normal numbers of chromosomes and no obvious large repetition/deletion fragments (Fig. 2F). These results provide a potential treatment for these patients in the future.

We found phenotypic variability among the affected individuals with *CDC20* mutations. In brief, the proband in family 1 and 2 had the phenotype of oocyte maturation arrest. Although the proband in family 3 had a slight delay in oocyte maturation, the ultimate phenotype in family 3 and 4 was characterized by fertilization failure. The proband in family 5 showed early embryonic arrest. It has been reported in mice that *Cdc20* has the highest expression in metaphase II (MII) oocytes compared to GV and MI oocytes as well as early embryos (Amanai et al., 2006), and this expression pattern was also observed in our qRT-PCR results in human oocytes and early embryos (Fig. S5). It is therefore likely that different amounts of CDC20 are needed at different stages of oocyte maturation, fertilization, and early embryo development. As for the mutations we identified, both family 1 and 2 had homozygous missense mutations, while families 3–5 harbored heterozygous compound mutations including one missense mutation and either a nonsense mutation or a frameshift mutation. The combinations of various types of mutations may result in different degrees of impairment of CDC20 and thus could lead to phenotypic variability.

Although CDC20 plays an important role in both mitosis and meiosis (Musacchio and Hardwick, 2002; Jones, 2011), all affected individuals in the study only exhibited the phenotype of female infertility without any abnormalities in somatic tissues or organs. This might due to the different thresholds of CDC20 amount required between mitosis and meiosis (Fig. S5). In addition, previous studies showed that *Cdc20* knockout mice were embryonic lethal, while *Cdc20* hypomorphic mice with graded reduction of CDC20 protein level from 60%–27% were healthy and had a normal lifespan compared to wild type mice, but only female hypomorphic mice with 27% CDC20 protein level were infertile or subfertile (Jin et al., 2010; Malureanu et al., 2010). In contrast, in mitosis even profound reduction of CDC20 levels to 10% of normal still supports the onset of anaphase and the completion of mitosis (Wolthuis et al., 2008). All of these results indicate that female infertility resulting from CDC20 reduction is dosage dependent and that mitosis is more tolerant than meiosis to CDC20 reduction.

In summary, we identified biallelic mutations in *CDC20* responsible for variable phenotypes of female infertility characterized by abnormalities in oocyte maturation, fertilization and early embryonic development and implicated cRNA injection strategy for a potential therapeutic treatment for these patients.

## Electronic supplementary material

Below is the link to the electronic supplementary material.

Supplementary material 1 (PDF 3777 kb)

## References

[CR1] Alazami AM, Awad SM, Coskun S, Al-Hassan S, Hijazi H, Abdulwahab FM, Poizat C, Alkuraya FS (2015). TLE6 mutation causes the earliest known human embryonic lethality. Genome Biol.

[CR2] Amanai M, Shoji S, Yoshida N, Brahmajosyula M, Perry AC (2006). Injection of mammalian metaphase II oocytes with short interfering RNAs to dissect meiotic and early mitotic events. Biol Reprod.

[CR3] Chen T, Bian Y, Liu X, Zhao S, Wu K, Yan L, Li M, Yang Z, Liu H, Zhao H (2017). A Recurrent Missense Mutation in ZP3 Causes Empty Follicle Syndrome and Female Infertility. Am J Hum Genet.

[CR4] Feng R, Sang Q, Kuang Y, Sun X, Yan Z, Zhang S, Shi J, Tian G, Luchniak A, Fukuda Y (2016). Mutations in TUBB8 and Human Oocyte Meiotic Arrest. N Engl J Med.

[CR5] Huang HL, Lv C, Zhao YC, Li W, He XM, Li P, Sha AG, Tian X, Papasian CJ, Deng HW (2014). Mutant ZP1 in familial infertility. N Engl J Med.

[CR6] Jin F, Hamada M, Malureanu L, Jeganathan KB, Zhou W, Morbeck DE, van Deursen JM (2010). Cdc20 is critical for meiosis I and fertility of female mice. PLoS Genet.

[CR7] Jones KT (2011). Anaphase-promoting complex control in female mouse meiosis. Results Probl Cell Differ.

[CR8] Li L, Fan L, Peng N, Yang L, Mou L, Huang W (2017). R383C mutation of human CDC20 results in idiopathic non-obstructive azoospermia. Oncotarget.

[CR9] Malureanu L, Jeganathan KB, Jin F, Baker DJ, van Ree JH, Gullon O, Chen Z, Henley JR, van Deursen JM (2010). Cdc20 hypomorphic mice fail to counteract de novo synthesis of cyclin B1 in mitosis. J Cell Biol.

[CR10] Musacchio A, Hardwick KG (2002). The spindle checkpoint: structural insights into dynamic signalling. Nat Rev Mol Cell Biol.

[CR11] Nasmyth K, Haering CH (2005). The structure and function of SMC and kleisin complexes. Annu Rev Biochem.

[CR12] Reis A, Madgwick S, Chang HY, Nabti I, Levasseur M, Jones KT (2007). Prometaphase APCcdh1 activity prevents non-disjunction in mammalian oocytes. Nat Cell Biol.

[CR13] Sang Q, Zhang Z, Shi J, Sun X, Li B, Yan Z, Xue S, Ai A, Lyu Q, Li W (2019). A pannexin 1 channelopathy causes human oocyte death. Sci Transl Med.

[CR14] Wolthuis R, Clay-Farrace L, van Zon W, Yekezare M, Koop L, Ogink J, Medema R, Pines J (2008). Cdc20 and Cks direct the spindle checkpoint-independent destruction of cyclin A. Mol Cell.

[CR15] Xu Y, Shi Y, Fu J, Yu M, Feng R, Sang Q, Liang B, Chen B, Qu R, Li B (2016). Mutations in PADI6 Cause Female Infertility Characterized by Early Embryonic Arrest. Am J Hum Genet.

