# Supplementary material for: Biallelic mutations in CDC20 cause female infertility characterized by abnormalities in oocyte maturation and early embryonic development
Source: Protein Cell. 2020 Jul 14;11(12):921–7. doi: 10.1007/s13238-020-00756-0 (PMC7719138; doi:10.1007/s13238-020-00756-0)
Supplement: Supplementary file 1 — Supplementary material 1 (PDF 3777 kb) [file 13238_2020_756_MOESM1_ESM.pdf]

Supplementary Materials and Methods

Clinical samples

Infertility-affected individuals and healthy controls were recruited from the Shanghai East Hospital affiliated with Tongji University, the Ninth Hospital affiliated with Shanghai Jiao Tong University, the Shanghai Ji Ai Genetics and IVF Institute affiliated with the Obstetrics and Gynecology Hospital of Fudan University, the Reproductive Medical Center of Xiangya Hospital affiliated with Central South University, and the Reproductive Medicine Center of the Shaanxi Maternal and Child Care Service Center. Peripheral blood samples were taken for DNA extraction.

Ovarian stimulation and embryo culture

The superovulation was performed by human chorionic gonadotropin (hCG) (2,000 IU; Lizhu Pharmaceutical Trading Co, China) triggered, then oocytes retrieval was scheduled at 34-36 hours after trigger and IVF or ICSI was performed 4-5 hours after oocytes retrieval.

For IVF, collected oocytes were incubated in human tubal fluid (HTF; Irvine Scientific, United States), supplemented with 10% serum substitute supplement (SSS; Irvine Scientific, United States), and 300,000 progressively motile spermatozoa and left overnight; For ICSI, denudated oocytes were injected with a single mechanically immobilized sperm and directly thereafter cultured in fertilization medium (HTF + 10% SSS). All embryos were maintained in Continuous Single Culture of HTF (Irvine Scientific, United States) and were incubated under oil at 37 ℃ and a 5% O_2 and 6%

CO_2 humidified incubators with 30 μL of culture media drop throughout the entire duration of *in vitro* culture.

Genetic studies

Genomic DNA was extracted from peripheral blood using the QIAamp DNA Blood Mini Kit (Qiagen). Whole-exome capture was performed using the SeqCap EZ Exome Kit (Roche), and sequencing analysis was compared with the human reference sequence (NCBI Genome build GRCh37). Variants were annotated with GRCh37 and the dbSNP (version 138), genome Aggregation Database (gnomAD), and Exome Aggregation Consortium (ExAC) databases along with our in-house exome database, and functional prediction was performed with the SIFT and MutationTaster programs. In patients from consanguineous families, homozygosity mapping was performed with Homozygosity Mapper(Brazas, et al., 2009). All candidate variants were screened with the following criteria: 1. variants with frequencies less than 0.1% in public databases, 2. variants predicted to be loss of function or to be damaging by SIFT or MutationTaster, and 3. variants inherited from both the father and mother if parents' blood samples were available. The candidate genes were confirmed by Sanger sequencing of the affected probands as well as their parents and unaffected sibling.

Expression vector construction

The full-length coding sequence of wild-type *CDC20* was amplified from the cDNA of abandoned control human MI oocytes and cloned into the pCMV6-empty vector. Site-directed mutagenesis was performed to introduce corresponding

mutations into the wild-type vector using the KOD-Plus Mutagenesis Kit (Toyobo) according to the manufacturer's protocol. The flag tag was inserted into the wild type and mutant vectors at the C-terminal for the localization experiment.

Cell culture and transfection

CHO cells were cultured in Dulbecco's Modified Eagle Medium supplemented with 10% fetal bovine serum and 1% penicillin/streptomycin (Gibco) and maintained in a humidified incubator at 37 ℃ with 5% CO_2. Wild-type and mutant plasmids of *CDC20* were transfected into CHO cells using the PolyJet *In Vitro* DNA Transfection Reagent (SignaGen) according to the manufacturer's instructions.

Generation of Lymphoblastoid Cell Lines

Lymphoblastoid cell lines (LCLs) were generated from the individual II-1 in family 3 and two independent controls by transformation with Epstein-Barr virus (EBV)-containing supernatant as previously described (Tosato and Cohen, 2007).Cell lines were confirmed by sequencing of the *CDC20* mutations before their use in experiments.

Western blotting

The CHO cells were incubated for 36 h after being transfected with *CDC20* wild-type and mutant plasmids. Antibodies against CDC20 (14866, CST) and Cyclin B1 (55004-1-AP, Proteintech) were used at 1:1,000 dilution, and antibody anti-vinculin (1:3,000 dilution, 13901, CST) was used as internal control. Goat anti-rabbit IgG (1:5,000 dilution, M21001, Abmart) and goat anti-mouse IgG (1:5,000 dilution, M21002, Abmart) were used as the secondary antibodies to detect the

primary antibodies. The blots were finally captured using ECL Western Blotting Substrate (Tanon) after incubation with the secondary antibodies.

***CDC20* expression analysis**

Total RNA from human GV, MI, and MII oocytes, day 3 embryos, blastocysts, other somatic tissues (including heart, liver, spleen, lung, kidney, brain, spinal cord, and testis), LCLs and granulosa cells (GC) of control and patients, wild-type and mutant CDC20 transfected CHO cells, were extracted with the RNeasy Mini Kit (Qiagen). Reverse transcription was performed with the PrimeScript RT Reagent Kit (Takara) according to the manufacturer’s instructions. Expression of *CDC20* was determined with specific primers (Table S3) and was normalized to the expression of human *GAPDH* (h*GAPDH*) or mouse *Gapdh* (m*Gapdh*) as the internal control.

Oocyte collection and microinjection

Germinal vesicle (GV) oocytes were collected from the ovaries of 7–8-week-old ICR mice. For long-term incubation, oocytes were cultured in Minimum Essential Medium (Gibco) with 10% fetal bovine serum and 1% penicillin/streptomycin (Gibco) in a humidified incubator at 37 ℃ with 5% CO_2. 200μM iso-butyl-methyl-xanthine (IBMX, Sigma) was used to prevent GV breakdown. For the localization experiment, wild-type and mutant FLAG-tagged *CDC20* cRNA were injected into GV oocytes. For collecting the MI oocytes, the injected GV oocytes were released into IBMX-free medium and subsequently fixed for immunofluorescence at 7 h after release. For the siRNA experiment, *Cdc20* 5'UTR siRNA (si*Cdc20*, 5'–UGU UCG GGA GAG CUG AGU ATT–3', 40 μM) was injected into GV oocytes and incubated for 24 h to knock

down the endogenous Cdc20 level, which led to MI arrest. For the rescue experiment, wild-type *CDC20* cRNA (750 ng/μl) and mutant *CDC20* cRNA (750 ng/μl) were injected into GV oocytes at 6 h after siRNA injection. The polar body extrusion rate was recorded at 12 h after being released into free medium.

Immunofluorescence

Oocytes were fixed and stained as previously described (Wu, et al., 2018). FLAG antibody (1:500 dilution, A9594, Sigma-Aldrich) was used for determining the CDC20 localization. MI oocytes were also stained with Crest antibody (1:500 dilution, HCT-0100, Immunovision) to label the kinetochores and Hoechst (1:700 dilution, 33342, BD) to label the DNA. Oocytes were mounted on glass slides and images were captured on a confocal laser-scanning microscope (Leica).

Preimplantation Genetic Screening of Blastocyst

Trophectoderm cells were aspirated into a biopsy pipette from the blastocyst embryo. Whole-genome amplification of the embryo biopsy sample was performed using the MALBAC WGA kit (Yikon Genomics, Shanghai, China) following the manufacturer’s instructions. The chromosomal copy number and large repetition/deletion fragments were analyzed as published previously (Zong, et al., 2012).

Supplementary figure legends

Figure S1. Homozygosity mapping of affected individuals. Homozygosity mapping of the affected individuals in consanguineous Families 1 and 2. Homozygous regions harboring the strongest signal are indicated in red. The asterisk (*) indicates the area where *CDC20* is located.

Figure S2. Analysis of CNV in probands of family 1 and family 2. (A) Results of the CNV analysis in individual II-1 of family 1. (B) Results for CNV analysis in individual II-1 of family 2. For A and B, the red head arrow indicates the *CDC20* region, the black dots indicate no copy number alterations in the *CDC20* region.

Figure S3. Quantitation of western blotting and relative expression of *CDC20* mRNA in LCLs and GCs of affected individuals and controls. (A) Quantitation of total CDC20 protein level in LCLs of two controls and individual II-1 in family 3 in Figure 1F. Quantitation was performed by measuring the band intensity of CDC20 relative to that of vinculin. (B) The relative expression of *CDC20* in LCLs of individual II-1 in family 3 and two independent controls. (C) The relative expression of *CDC20* in GCs of individual II-1 in family 4 and one control. For B and C, the

relative expression was measured by qRT-PCR and normalized to h*GAPDH*, three independent experiments were performed, the bars show the mean of three separate measurements, and error bars denote standard deviations. **, $p <0.01$, *** $p < 0.001$, ****, $p <0.0001$, ns, not significant.

Figure S4. Relative mRNA expression of *CDC20* mutations in transfected CHO cells. The relative expression of *CDC20* mRNA in transfected CHO cell as measured by qRT-PCR and normalized to mouse *Gapdh* mRNA. The bars show the mean of three separate measurements, and error bars denote standard deviations. * $p<0.05$, ** $p < 0.01$.

Figure S5. Relative expression level of *CDC20* mRNA in different stages of human oocytes, embryos and several somatic tissues. The relative expression of *CDC20* mRNA in different stages of human oocytes, early embryos, and several somatic tissues as measured by qRT-PCR and normalized to h*GAPDH* mRNA. The bars show the mean of three separate measurements, and error bars denote standard deviations.

References

Brazas MD, Yamada JT, Ouellette BF. (2009). Evolution in bioinformatic resources: 2009 update on the Bioinformatics Links Directory. *Nucleic Acids Res* 37: W3-5.

Tosato G, Cohen JI. (2007). Generation of Epstein-Barr Virus (EBV)-immortalized B cell lines. *Current protocols in immunology* Chapter 7: Unit 7.22.

Wu T, Lane SIR, Morgan SL, Jones KT. (2018). Spindle tubulin and MTOC asymmetries may explain meiotic drive in oocytes. *Nature communications* 9: 2952.

Zong C, Lu S, Chapman AR, Xie XS. (2012). Genome-wide detection of single-nucleotide and copy-number variations of a single human cell. *Science* 338: 1622-1626.

Table S1. Oocytes and embryos characteristics of IVF/ICSI attempts of five affected individuals

Individual	Age (Years)	Duration Of Infertility (Years)	IVF/ICSI Attempts	Total No. of Oocytes	GV Oocytes	MI Oocytes	PB1 Oocytes	Oocytes with Abnormal Morphology	Fertilized Oocytes	Embryos Arrested at Early Stage	Usable Embryos	Outcome of Embryo Transfer
II-1 in family 1	36	3	IVF ×2	13	0	2	10	1	7	4	3	failed
			ICSI ×1	11	5	5	1	0	1	1	0	/
II-1 in family 2	/	6	ICSI ×2	19	0	19	0	0	/	/	/	/
II-1 in family 3	35	9	ICSI ×2	20	0	7	10	3	2	2	0	/
II-1 in family 4	34	7	ICSI ×2	11	0	2	9	0	0	0	0	/
II-1 in family 5	35	9	IVF ×1	3	0	0	3	0	2	1	0	/

IVF, *in vitro* fertilization; ICSI, intracytoplasmic sperm injection; GV, germinal vesicle; MI, metaphase I; PB1, the first polar body; / not available

Table S2. Overview of the *CDC20* mutations observed in the five families

Family	Genomic Position on Chr.1 (bp)	cDNA Change	Protein Change	Mutation Type	SIFT[a]	MutTas[a]	gnomAD_ea[b]	EXAC_ea[b]
1	43,825,990	c.683A>G	p.Tyr228Cys	missense	D	D	0	NA
2	43,827,978	c. 1316T>G	p.Leu439Arg	missense	D	D	NA	NA
3	43,825,756	c.544C>T	p.Arg182*	stop gain	NA	D	NA	NA
	43,826,520	c.965G>A	p.Arg322Gln	missense	T	D	9.021×10^{-4}	4.622×10^{-4}
4	43,826,229_43,826,230	c.813_814ins AGTG	p.Gly272Serfs*24	frameshift insertion	NA	D	NA	NA
	43,826,520	c.965G>A	p.Arg322Gln	missense	T	D	9.021×10^{-4}	4.622×10^{-4}
5	43,826,520	c.965G>A	p.Arg322Gln	missense	T	D	9.021×10^{-4}	4.622×10^{-4}
	43,826,889_43,826,892	c.1176_1179del TCTG	p.Cys392*	frameshift deletion	NA	D	NA	NA

[a] Mutation assessment by SIFT and MutationTaster (MutTas). T, tolerated; D, damaging. NA, not available

[b] Frequency of corresponding mutations in the East Asian population of gnomAD and ExAC Browser. NA, not available

Table S3. List of primers used in real-time PCR

**Primer name**	**Sequence (5'-3')**
CDC20-RT-F	CGGAAGACCTGCCGTTACATTC
CDC20-RT-R	CAGAGCTTGCACTCCACAGGTA
h*GAPDH*-RT-F	CAAATTCCATGGCACCGTCA
h*GAPDH*-RT-R	GGCAGAGATGATGACCCTTT
m*Gapdh*-RT-F	CATCACTGCCACCCAGAAGACTG
m*Gapdh*-RT-R	ATGCCAGTGAGCTTCCCGTTCAG

Family 1

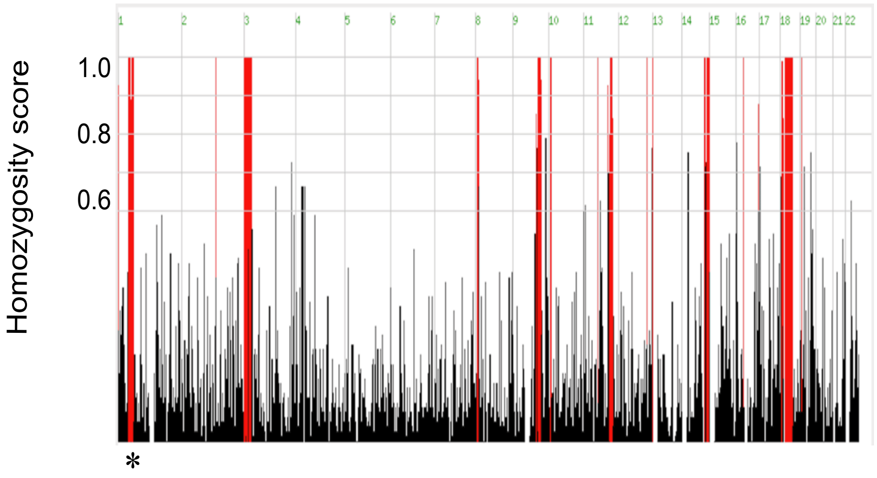

Family 2

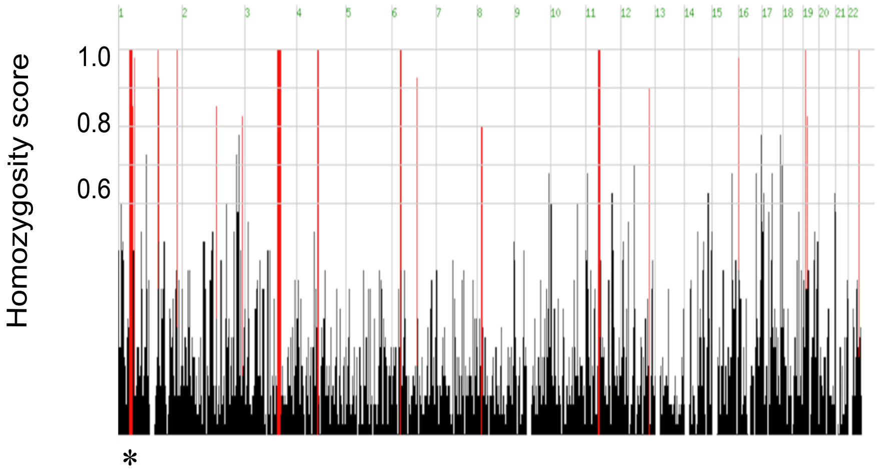

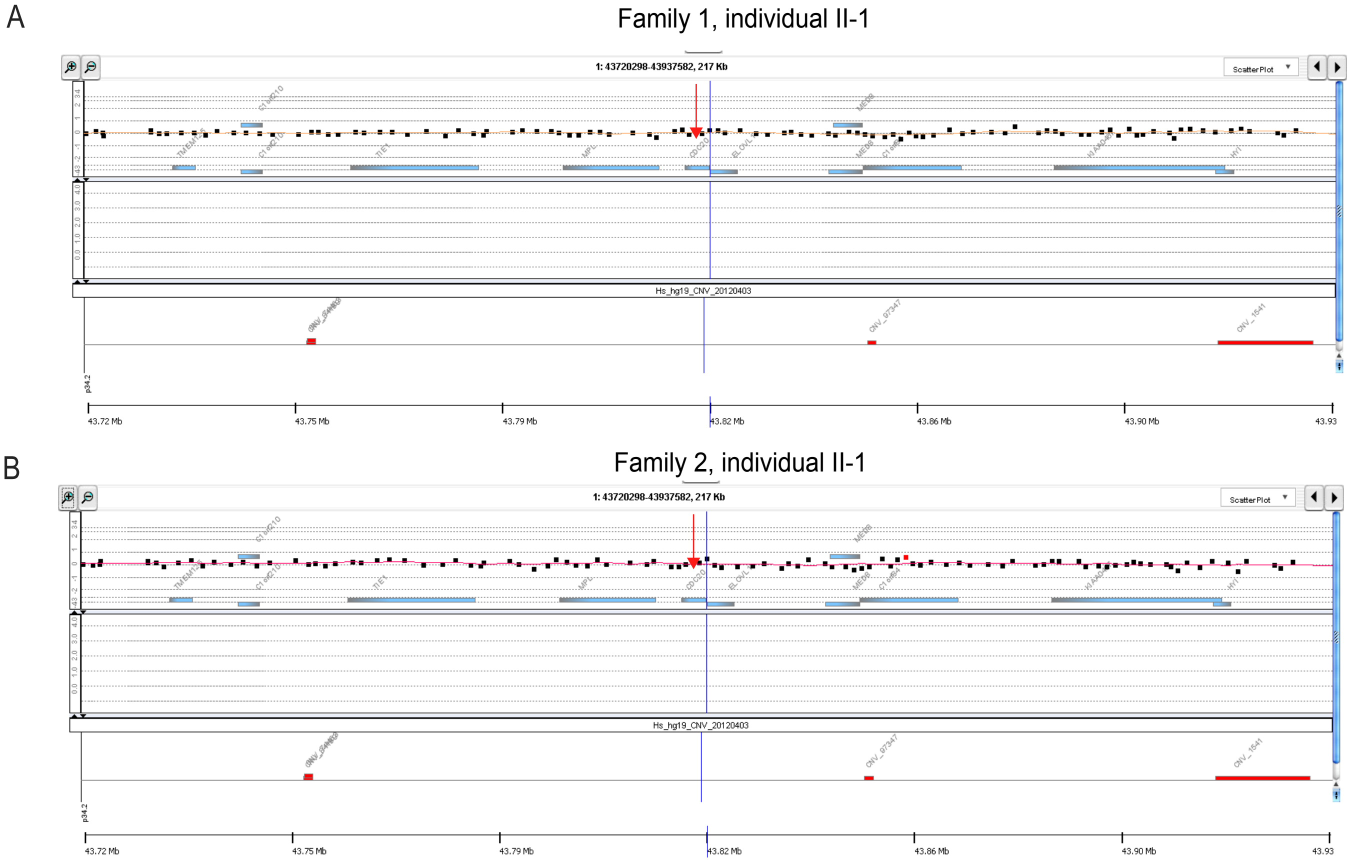
A
Family 1, individual II-1
1: 43720298-43937582, 217 Kb
Scatter Plot
Hs_hg19_CNV_20120403
43.72 Mb
43.75 Mb
43.79 Mb
43.82 Mb
43.86 Mb
43.90 Mb
43.93
B
Family 2, individual II-1
1: 43720298-43937582, 217 Kb
Scatter Plot
Hs_hg19_CNV_20120403
43.72 Mb
43.75 Mb
43.79 Mb
43.82 Mb
43.86 Mb
43.90 Mb
43.93

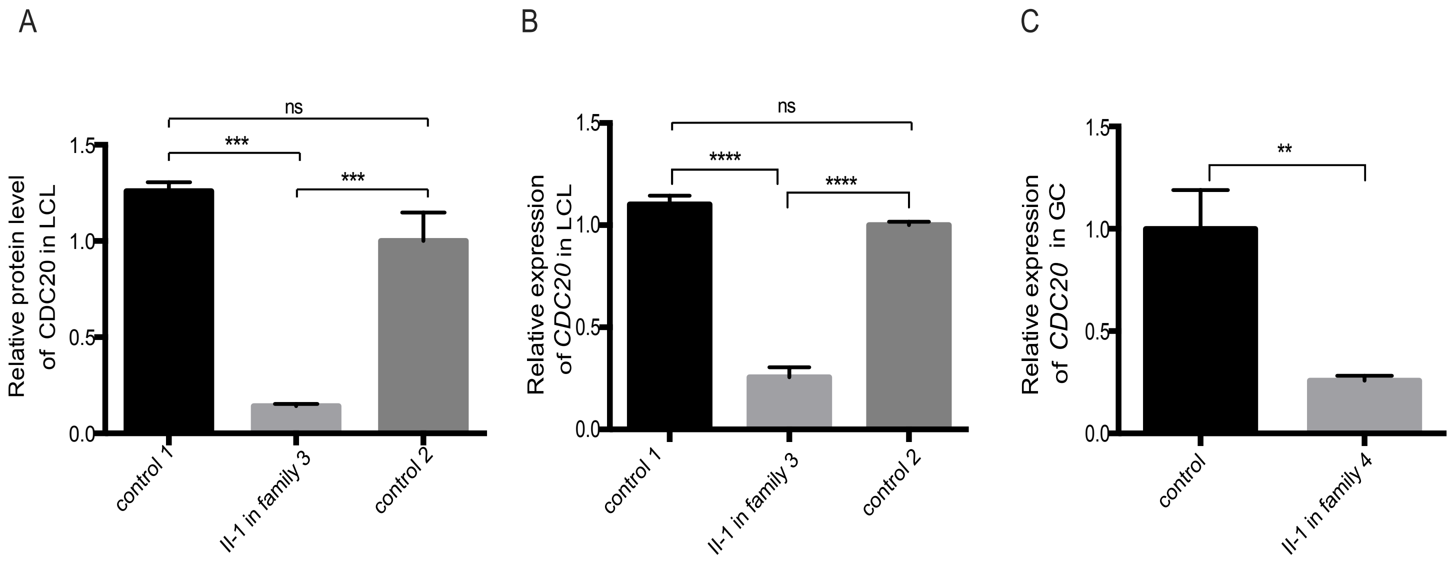

A
ns
***
***
1.5
1.0
0.5
0.0
Relative protein level
of CDC20 in LCL
control 1
II-1 in family 3
control 2
B
ns
****
****
1.5
1.0
0.5
0.0
Relative expression
of CDC20 in LCL
control 1
II-1 in family 3
control 2
C
**
1.5
1.0
0.5
0.0
Relative expression
of CDC20 in GC
control
II-1 in family 4

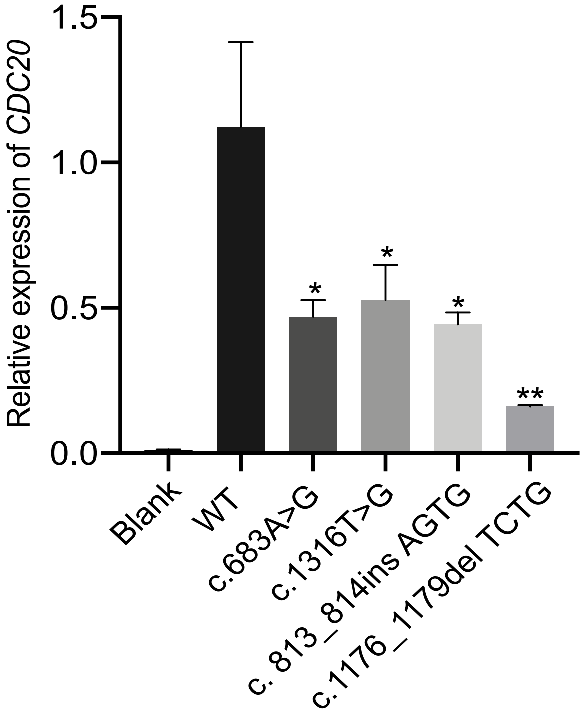

Relative expression of *CDC20*
1.5
1.0
0.5
0.0
*
*
*
**
Blank
WT
c.683A>G
c.1316T>G
c. 813_814ins AGTG
c.1176_1179del TCTG

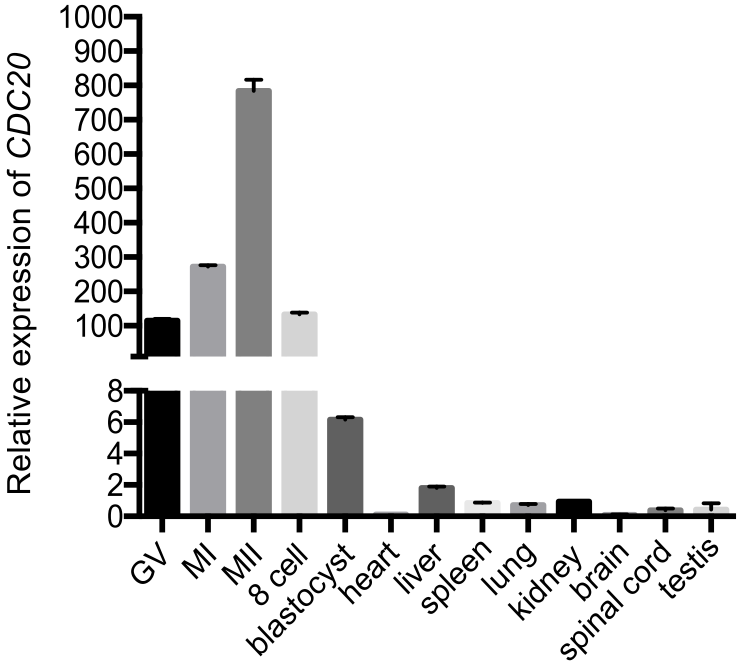

Relative expression of *CDC20*
1000
900
800
700
600
500
400
300
200
100
8
6
4
2
0
GV
MI
MII
8 cell
blastocyst
heart
liver
spleen
lung
kidney
brain
spinal cord
testis
